# Tafenoquine succinate inhibits the growth of the equine piroplasmosis hemoparasites *Theileria equi* and *Babesia caballi*

**DOI:** 10.1186/s13071-026-07262-y

**Published:** 2026-01-27

**Authors:** Natalia N. Cardillo, Nicolas F. Villarino, Lowell S. Kappmeyer, Chungwon J. Chung, Carlos E. Suarez, Reginaldo G. Bastos

**Affiliations:** 1 Department of Veterinary Microbiology and Pathology, Washington State University, Pullman, WA USA; 2 Animal Disease Research Unit, USDA-ARS, Pullman, WA USA; 3 Department of Veterinary Clinical Sciences, College of Veterinary Medicine, Washington State University, Pullman, USA

**Keywords:** Therapeutics, Horses, Tafenoquine succinate, Imidocarb dipropionate

## Abstract

**Background:**

Equine piroplasmosis (EP) is a tick-borne disease of equids caused by the intraerythrocytic apicomplexan parasites *Theileria equi*, *Babesia caballi* and the recently identified *Theileria haneyi*. Acute cases can be severe, with anemia, jaundice, abortion or sudden death. Survivors remain lifelong carriers, serving as reservoirs for tick-borne and iatrogenic transmission. No vaccines are currently available, and control strategies rely heavily on accurate diagnostics and chemotherapeutic intervention. Imidocarb dipropionate (ID) is the current standard of care for both acute treatment and radical cure. However, growing concerns regarding ID-resistant parasite strains and its associated toxicity have highlighted the urgent need for novel, safer and more effective antiparasitic agents. Here, we assessed the in vitro efficacy of tafenoquine succinate (TFQ), a synthetic 8-aminoquinoline with broad antiparasitic activity, against *T. equi* and *B. caballi* as a potential treatment for equine piroplasmosis.

**Methods:**

The effect of TFQ on *T. equi* and *B. caballi* was evaluated in vitro in parasite cultures. The percentage of parasitized erythrocytes was measured by flow cytometry, and the effect of TFQ on parasite growth was compared to that of ID. TFQ toxicity on horse peripheral blood mononuclear cells (PBMCs) was assessed via a colorimetric metabolic assay.

**Results:**

TFQ reduced *T. equi* parasitemia in a dose-dependent manner, matching ID efficacy at 72 h. For *B. caballi*, TFQ had no effect at 5–10 µM but inhibited growth at 15 µM, similar to the results obtained with ID. TFQ exhibited approximately threefold greater potency against *T. equi* [half-maximal inhibitory concentration [IC_50_] 5.90 μM, 95% confidence interval (CI) 4.99–5.96; 99% inhibitory concentration (IC_99_) 60.74 μM, 95% CI 37.41–113.3] compared to *B. caballi* [IC_50_ 14.5 μM, 95% CI 13.81–15.23; IC_99_ 20.44 μM, 95% CI 17.77–28.84]. The narrower confidence intervals for *T. equi* suggest a more consistent antiparasitic response across replicates. Cytotoxicity assays showed no toxic effects on equine PBMCs at 2.5–5 μM (*P* > 0.05), while concentrations ≥ 10 μM indicated potential toxicity. These findings suggest that TFQ selectively targets parasites over host cells, supporting its therapeutic potential.

**Conclusions:**

TFQ significantly inhibited *T. equi* and *B. caballi* growth at doses tolerated by equine PBMCs, supporting its potential as an alternative treatment for EP and warranting further in vivo study.

## Background

Equine piroplasmosis (EP) is a significant tick-borne disease that affects horses worldwide, caused by the protozoan apicomplexan hemoparasites *Theileria equi*, *Babesia caballi* and the recently identified *Theileria haneyi* [1, 2]. The disease poses a serious threat to equine health and causes significant economic losses in the equine industry due to its impact on animal performance and restriction on international movement [3, 4]. In addition to tick transmission, iatrogenic and transplacental transmissions of EP parasites have also been reported [5, 6]. Currently, no vaccines are available against EP, and control relies on diagnostics testing, antiparasitic drugs and supportive treatments [1]. Current antiparasitic treatment options for EP are limited, with imidocarb dipropionate (ID) being the drug of choice [7]. However, ID-linked toxicity in horses, lack of effectiveness against *T. haneyi* and drug-resistant field isolates of *T. equi* and *B. caballi* have been documented [8]. Additionally, a previous study demonstrated that co-infection of *T. haneyi* and *T. equi* reduces the efficacy of ID against *T. equi* [9]. This is particularly concerning, as ID remains the primary drug used globally to manage the clinical signs of acute disease and to achieve the complete cure of EP [10, 11]. Taken together, these factors underscore the urgent need for new, safer and more effective therapeutic strategies to control the causative agents of EP [8, 11].

Tafenoquine succinate (TFQ) is an 8-aminoquinoline compound discovered by the Walter Reed Army Institute of Research in 1978 as a potential replacement for primaquine in the treatment and prevention of malaria caused by *Plasmodium* parasites [12, 13]. TFQ is effective not only against *Plasmodium falciparum* but also in achieving the radical cure of *Plasmodium vivax* malaria, owing to its pharmacological properties and long half-life [14, 15]. In addition, recent studies have demonstrated that TFQ is effective against *Babesia microti*, one of the major causative agents of human babesiosis, an emerging tick-borne disease in the USA, Europe and Asia [16, 17]. While the mechanism of action of TFQ is not fully understood, it is known that the drug can disrupt mitochondrial function [18, 19], inhibit hematin polymerization [20] and induce oxidative stress [15, 21]. These factors can contribute to the drug’s efficacy against various *Plasmodium* species and parasite stages, including pre-erythrocytic (liver stages), erythrocytic (asexual stages) and gametocyte stages, thereby helping to provide prophylactic protection, prevent relapses and achieve radical cure [19, 22, 23]. Safety studies have demonstrated that TFQ is well tolerated when administered orally to humans and laboratory animals [24]. Considering both the safety and efficacy data of TFQ, the US Food and Drug Administration (FDA) approved the drug in 2018 for prophylactic use and the radical cure of human malaria [25]. While generally well-tolerated, TFQ can cause asymptomatic hemoglobin decline and is contraindicated in glucose-6-phosphate dehydrogenase (G6PD)-deficient individuals [26, 27]. Although G6PD deficiency affects approximately 400 million people globally, the alteration is considered rare or non-existent in agricultural animal species [28]. To the best of our knowledge, no studies have been so far reported on the effect of TFQ on *Theileria* and *Babesia* species that affect agricultural animals.

Considering the broad efficacy of TFQ against apicomplexan parasites, including *Plasmodium* and *Babesia* species, we evaluated the in vitro inhibitory effect of the drug on the growth of *T. equi* and *B. caballi*. This comparative efficacy study between tafenoquine and imidocarb dipropionate aimed to evaluate TFQ as a potential new treatment for EP, with the goal of overcoming the limitations of currently approved drugs.

## Methods

### Drug compounds

Lyophilized TFQ, with ≥ 95% purity determined by high-performance liquid chromatography (HPLC), was purchased from Millipore-Sigma (catalog number TSML0396; St. Louis, MO, USA). The compound was suspended in dimethyl sulfoxide (DMSO), following the manufacturer’s recommendations. ID (VETRANAL™; Supelco®, Sigma-Aldrich Chemie GmbH, Buchs, Switzerland) was used as a reference compound in the in vitro inhibition assays for *T. equi* and *B. caballi*, following the same protocol described below for TFQ. The purity of ID was determined to be > 98% by proton-nuclear magnetic resonance spectroscopy and HPLC, according to the certificate of analysis. TFQ and ID were diluted in 100% DMSO to prepare stock solutions, which were stored at room temperature until use. Working solutions were freshly prepared in parasite culture medium on each test day prior to being added to the parasite cultures.

### *Theileria equi *and* B. caballi* in vitro culture

The *T. equi* Florida isolates and *B. caballi* Puerto Rico isolates used in this study were maintained in long-term microaerophilic stationary-phase cultures at 37° C under atmospheric condition of 5% CO_2_, 5% O_2_ and 90% N_2_, as previously described [29–31]. Both parasites were cultured in 24-well culture plates, containing 20% (v:v) red blood cells (RBCs) in 1 milliliter per well of HL-1 culture medium (pH 7.2).

### Parasite growth inhibition assay

*Theileria equi* and *B. caballi* cultures were initiated at parasitized erythrocyte percentages (PPE) of 1% and 0.5%, respectively. Parasites were cultured in media containing TFQ and ID at concentrations of 5, 10, and 15 µM, diluted in 100% DMSO. All assays were performed in triplicate wells (technical replicates) for each drug concentration and corresponding controls over an 8-day period. Although only a single experiment was conducted, the triplicates provide an indication of variability within the assay. Cultures maintained in drug-free medium and uninfected RBCs served as positive and negative controls, respectively. Drug-containing media were replaced daily for the first 3 days of culture. At 72 h after the final treatment, the medium in all culture wells was replaced with fresh drug-free medium, and 100 μl of fresh RBCs was added to each well. Only fresh media (drug-free) was replaced daily for 4 more days to determine if the parasite cultures were viable and could continue growing in the absence of the drug. Parasitemia level was assessed daily using flow cytometry, between days 1 and 8, as described below.

### Determination of the 50% and 90% inhibitory concentration for TFQ

Cultures of both *T. equi* and *B. caballi* were initiated at 1% PPE. *Theileria equi* was treated with TFQ at concentrations of 1.25, 2.5, 3.75, 6.25, 7.5, 12.5, 15 and 25 μM, while *B. caballi* was treated with concentrations of 2.5, 3.75, 6.25, 7.5, 12.5, 15, 25 and 35 μM. All experiments were conducted in triplicate wells (technical replicates) for each concentration, including the corresponding controls. The drug-containing medium was replaced daily during 72 h (3 days) of culture, after which PPE was assessed by flow cytometry. In both experiments, DMSO-treated parasites (without drug) served as positive controls, and uninfected equine RBCs served as negative controls.

### Flow cytometric analysis

Parasitized erythrocyte percentages were determined by flow cytometry analysis after staining the parasite cultures with hydroethidine (HE), as previously described [4, 32]. Briefly, 5-µl aliquots of the cultures were collected from the bottom of the wells and suspended in 150 l of phosphate-buffered saline (PBS) at pH 7.2. The sample was then centrifuged at 450 *g* for 1 min at 4 ℃. This procedure was repeated twice, using the same buffer. The pellet was then resuspended in 200 μl of HE at a concentration of 25 μg/μl (Invitrogen, Thermo Fisher Scientific, Waltham, MA, USA) and incubated at 37 ℃ in 5% CO_2_ atmosphere for 20 min in the dark. Following incubation, the cells were washed twice with 200 μl of PBS to remove excess HE. The supernatant was then discarded, and the cell pellet was resuspended in 200 μl of fresh PBS. The resuspended cells were analyzed by flow cytometry using a Guava® easyCyte flow cytometer (Luminex Corp., Austin, TX, USA) at a ratio of 800–1000 cells/µl with 20,000 events collected. The results were analyzed by FCS Express v6 flow cytometry software (De Novo Software, Glendale, CA, USA). Uninfected horse RBCs were used as a negative control for the flow cytometric analysis.

### PBMC cytotoxicity assay

Cytotoxicity assays were conducted to evaluate the potential effects of TFQ on the viability of equine PBMCs, which were used as surrogates for nucleated vertebrate host cells. The assays were performed in in vitro cultures using 2.5, 5, 10, 15, 20 and 50 µM of TFQ. PBMC viability was assessed by monitoring cellular metabolic activity using a colorimetric WST-1 assay, as previously described [4]. Briefly, peripheral blood was collected from healthy horses via jugular venipuncture into Vacutainer® tubes containing acid citrate dextrose (ACD) (Becton, Dickinson and Company, Franklin Lakes, NJ, USA). PBMCs were isolated according to standard procedure using Histopaque® density gradient cell separation medium (Sigma-Aldrich, St. Louis, MO, USA). The cells were plated at a density of 2 × 10^5^ cells/well in 96-well plates in complete Dulbecco’s Modified Eagle Medium (cDMEM) (Sigma-Aldrich), containing 10% fetal bovine serum, 24 mM HEPES, 2 mM L-glutamine, 100 IU/ml penicillin and 100 µg/ml streptomycin. The cells were then incubated with the respective TFQ concentration. Cell proliferation reagent WST-1 (catalog number 05 015 944 001; Roche Applied Science, Penzberg, Germany) was added at 24-, 48- and 72-h post-exposure, according to the manufacturer’s instructions. Absorbance was measured at 440 nm using an enzyme-linked immunosorbent assay (ELISA) plate reader 4 h after adding WST-1. Negative controls included cells cultured in cDMEM without TFQ, as well as cells treated with DMSO alone (1:400 dilution, equivalent to the maximum volume used for TFQ compound dilutions). As a positive control for cytotoxicity, PBMCs were treated with 5 µg/ml concanavalin A (Con A) diluted in cDMEM.

### Specific selectivity index

The selectivity of TFQ against *T. equi* or *B. caballi* cells in comparison to mammalian cells (equine PBMCs) was assessed at the respective IC_50_ (concentration that inhibits parasite growth by 50%) using the standard formula described by Ndjakou Lenta et al. [32]: specific selectivity index (SSI) = IC_50_ (parasite)/CC_50_ (host cells), where CC_50_ (50% cytotoxic concentration) represents the concentration that reduces host cell viability by 50% [32].

### Statistical analysis

Parasitized erythrocyte percentages were assessed daily and comparisons among TFQ-treated cells, ID-treated cells and untreated control cells were performed using the Kruskal–Wallis nonparametric test. The drug concentrations required to determine the IC_50_ relative to the untreated control and the concentrations needed to achieve 99% inhibition (IC_99_) were estimated for TFQ using nonlinear regression analysis.

The normality of PBMC viability data was assessed using the Shapiro–Wilk test. As the data were not normally distributed and measurements were repeated on the same animals, the Friedman test was used to evaluate differences among TFQ concentrations at each time point. Significant differences were further analyzed using Dunn’s post hoc test with Bonferroni correction for multiple comparisons. Data are presented as mean ± standard deviation (SD), and a *P*-value < 0.05 was considered statistically significant. GraphPad Prism v7 software for Windows (GraphPad Software, Inc., San Diego, CA, USA) was used for the statistical analysis. The significance level was set at *P*  < 0.05.

## Results

We first evaluated the inhibitory effect of TFQ and ID on *T. equi* cultures to determine the initial susceptibility profile across a range of drug concentrations. Data are presented as mean ± SD of triplicate wells from a single experiment. While only one biological replicate was performed, the technical replicates allow assessment of intra-assay variability.

### TFQ exposure significantly inhibits the in vitro growth of *T. equi*

**Fig. 1:**
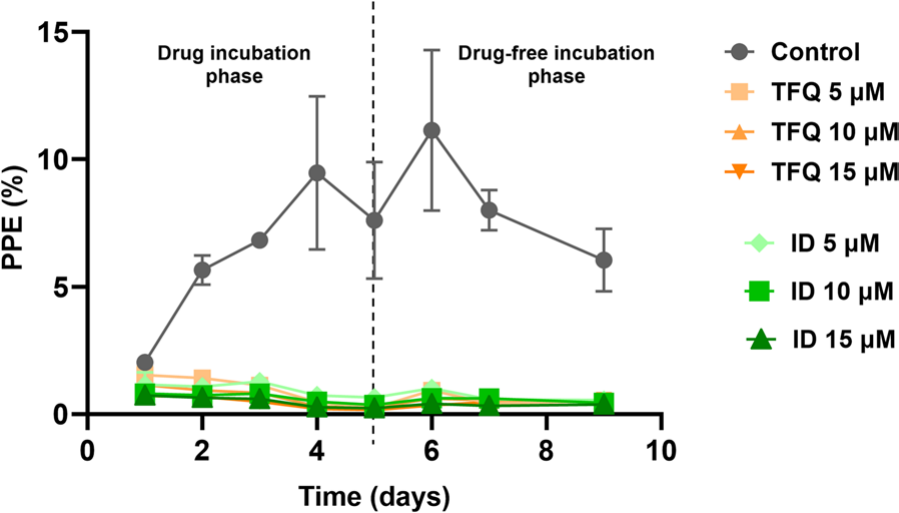
Mean percentage (±  standard deviation) of *Theileria equi*-parasitized erythrocytes in triplicate in vitro cultures following treatment with varying concentrations of TFQ and ID. Data are presented for the initial 3-day treatment period and the subsequent 5-day drug-free incubation period (days 4–8). ID, Imidocarb dipropionate; PPE, parasitized erythrocyte percentage; TFQ, tafenoquine succinate

**Fig. 2:**
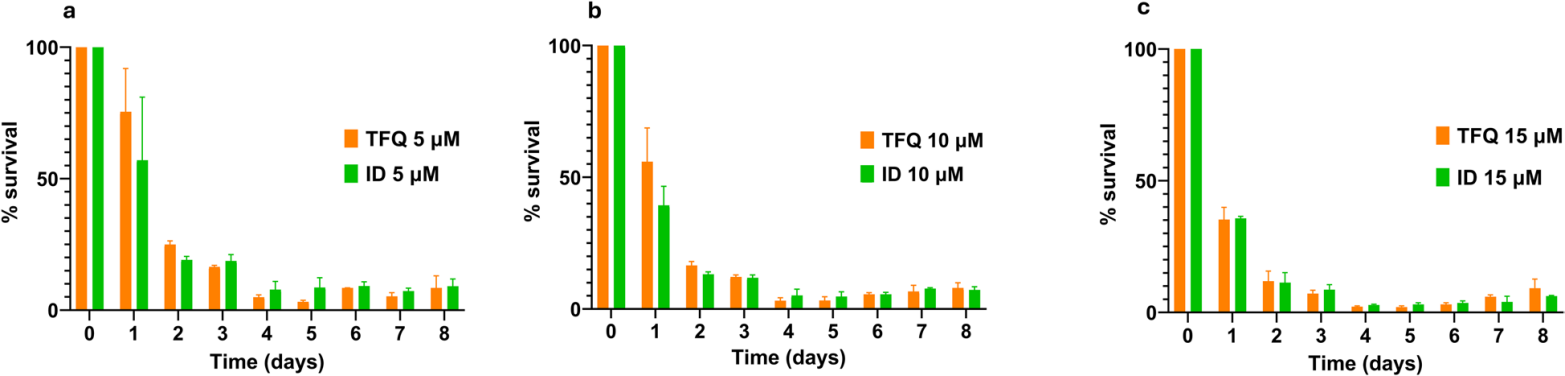
Mean percentage (± standard deviation) of *Theileria equi* survival in triplicate in vitro cultures after exposure to different concentrations of TFQ and ID: 5 µM (**a**), 10 µM (**b**) and 15 µM (**c**) of TFO or ID. Parasites were treated for an initial 3-day period with drugs, followed by a 5-day drug-free incubation phase (days 4–8). ID, Imidocarb dipropionate; TFQ, tafenoquine succinate

Tafenoquine exposure led to a progressive and consistent reduction in the *T. equi* PPE in a dose-dependent manner, resulting in significantly lower parasitemia compared to the untreated control (Fig. 1). Moreover, TFQ demonstrated efficacy comparable to that of ID at 72 h post-treatment across all tested concentrations, with no significant differences (*P* > 0.05) in inhibitory effect observed over time (Fig. 2a–c). During the 5-day drug-free incubation following the 3-day treatment period, a minimal parasite survival persisted, which fluctuated between 1.5% and 2% at different time points, but overall remaining at < 10%. The absence of complete parasite elimination, similar to what was observed with ID, may indicate a parasitostatic effect or suggest that higher doses could be required to achieve full elimination. The results of in vitro growth of *T. equi* during a 3-day drug treatment period with either TFQ or ID, followed by a 5-day drug-free incubation period, are shown in Fig. 1 and Fig. 2a–c.

Standard deviations for *T. equi* parasitemia were generally low across treatments and time points, indicating consistent and reliable measurements. Control samples showed moderate variability at some days, likely reflecting natural biological fluctuations. In contrast, TFQ-treated groups exhibited low variability overall (mostly < 0.3), especially at 5 and 10 µM, supporting stable and reproducible parasite growth inhibition. ID treatments showed similar low variability, with SD values < 0.3, reinforcing uniform and consistent effects across replicates. This controlled variability strengthens confidence in the observed differences between treatments and supports the robustness of the conclusions.

Given the variable drug susceptibility reported between *T. equi* and *B. caballi*, we next examined whether the inhibitory patterns observed in *T. equi* were conserved in *B. caballi* under identical experimental conditions.

### TFQ significantly suppress *B. caballi* growth

During the first 72 h, the growth of *B. caballi* was significantly inhibited by ID at all tested concentrations. In contrast, TFQ did not show any inhibitory effect at 5 µM and 10 µM when compared with the untreated control group throughout the 9-day observation period. For example, at day 7, parasitemia in the control group reached 8.48, while in the TFQ 5 µM and 10 µM groups it was 7.99 and 6.56, respectively. Although there is a slight numerical reduction, these decreases were not substantial and fell within a similar range considering the variability observed.

**Fig. 3:**
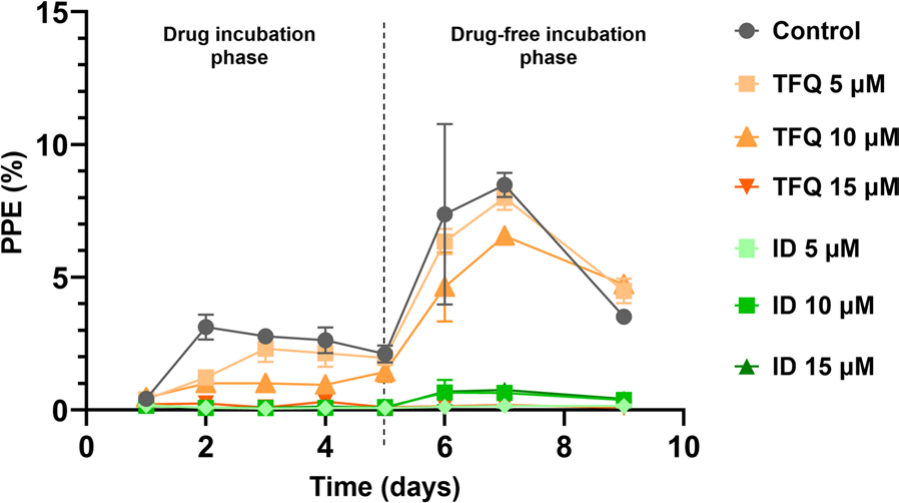
Mean percentage (± standard deviation) of *Babesia caballi*-parasitized erythrocytes in triplicate in vitro cultures following exposure to varying concentrations of TFQ and ID. Parasites were treated for an initial 3-day period, followed by a 5-day drug-free incubation phase (days 4–8). ID, Imidocarb dipropionate; PPE, parasitized erythrocyte percentage; TFQ, tafenoquine succinate

**Fig. 4:**
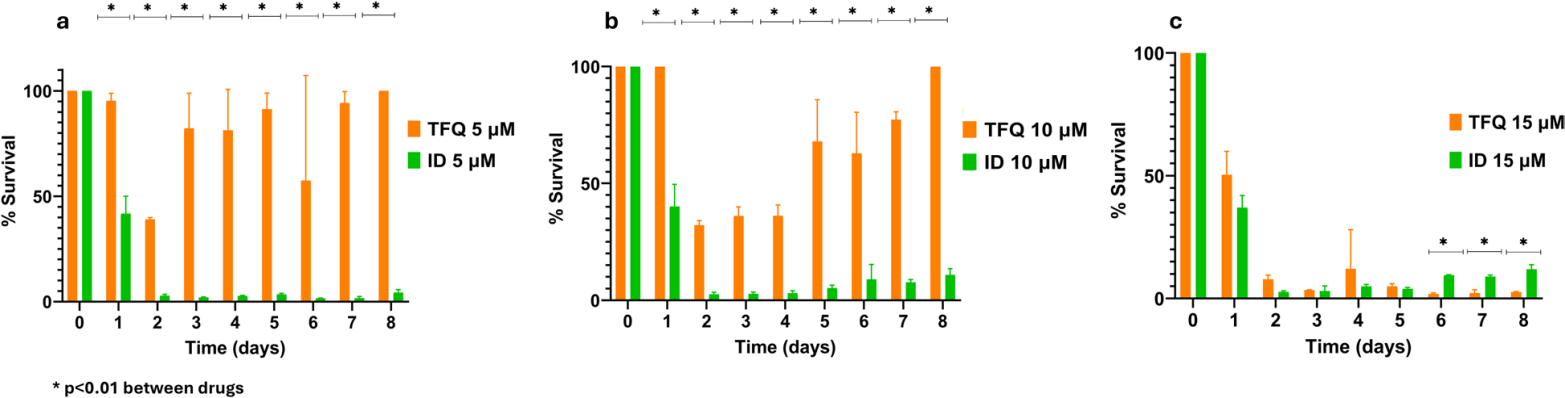
Mean percentage (± standard deviation) of *Babesia caballi* survival in triplicate in vitro cultures after exposure to different concentrations of TFQ and ID: 5 µM (**a**), 10 µM (**b**) and 15 µM (**c**). Parasites were treated for an initial 3-day period, followed by a 5-day drug-free incubation phase (days 4–8). ID, Imidocarb dipropionate; TFQ, tafenoquine succinate

However, at 15 µM, TFQ demonstrated a significant inhibitory effect on the growth of the parasite. Across the observed days, TFQ 15 µM reduced *B. caballi* parasitemia by 57–98% relative to the untreated control, with parasitemia values approaching zero after day 3. (Figs. 3, 4a–c). At 72 h post-treatment, TFQ showed limited efficacy against *B. caballi* at both 5 µM and 10 µM, with parasite growth rates similar to those observed in untreated control cultures. In contrast, ID exhibited significantly higher inhibitory activity at these concentrations, which was sustained throughout the observation period (*P* < 0.01). At a concentration of 15 µM, both TFQ and ID demonstrated significant efficacy against *B. caballi* beginning on the second day of treatment, with parasitemia reduced to 0.093–0.08%, respectively, during the 3-day treatment period, compared to 3.12% in the untreated controls (*P* < 0.05 at all time points). No significant differences were observed between these two groups through day 5. After this point, parasitemia in TFQ-treated cultures continued to decline, whereas recrudescence was observed in ID-treated cultures. Specifically, after ID treatment, the PPE showed a minimal rebound ranging from approximately 1.5% to 2%, whereas TFQ-treated cultures maintained parasitemia at < 1% throughout the drug-free incubation period. These results highlight the sustained inhibitory effect of TFQ compared to ID during the drug-free phase. Figures 3 and 4a–c, show the results of *B. caballi* in vitro growth during a 3-day exposure to either TFQ or ID, followed by a 5-day drug-free incubation period.

Standard deviations for *B. caballi* parasitemia were generally low to moderate across treatments and time points, indicating consistent and reproducible measurements. Control and TFQ treatments at 5 and 10 µM showed similar variability (most SD values < 0.5), supporting stable parasite growth comparable between these groups. At 15 µM TFQ, variability decreased alongside strong parasitemia suppression, while ID treatments consistently exhibited low variability (SD values < 0.1), reflecting uniform and effective parasite inhibition. This low variability strengthens confidence in the observed treatment effects and the conclusions drawn.

To quantify the inhibitory effects observed in the preceding experiments, we next calculated the IC_50_ and IC_99_ values for each compound. These parameters provide a more precise comparison of drug potency and allowed us to evaluate the magnitude of species-specific differences*.*

### Distinct TFQ potency and dose–response in *T. equi *than against *B. caballi*

**Fig. 5:**
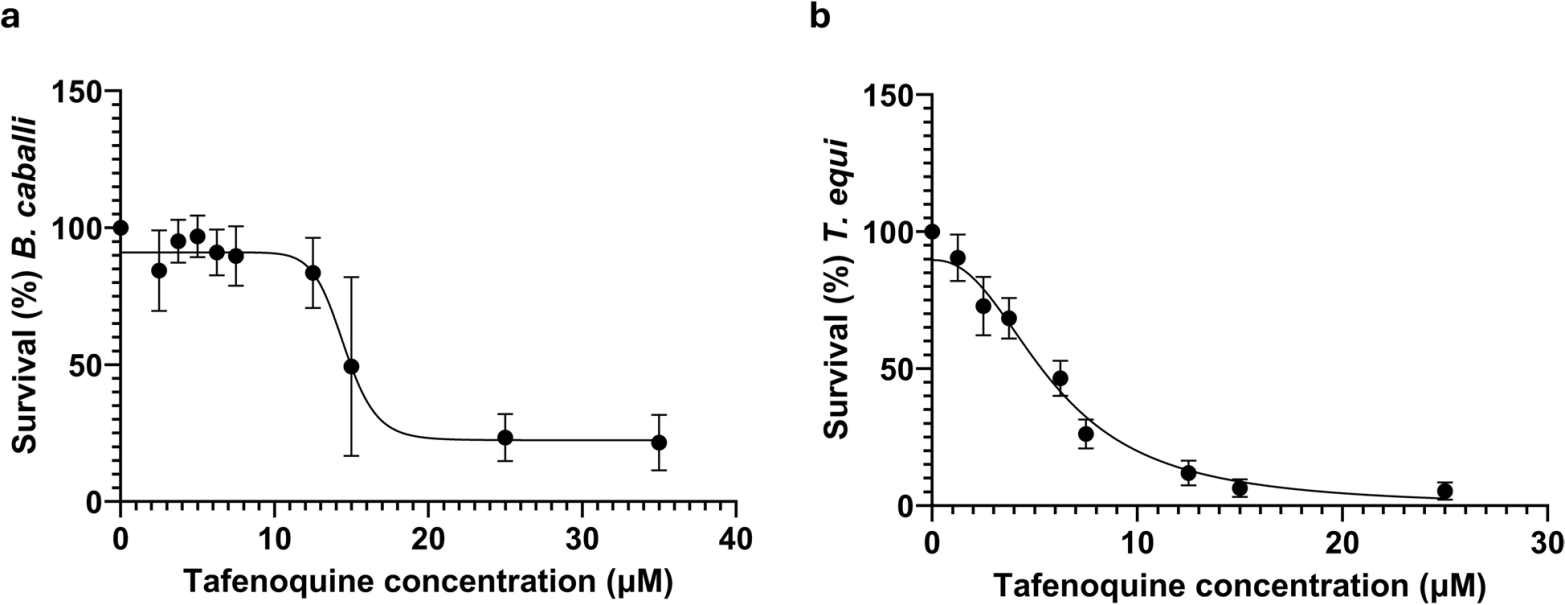
Potency of TFQ against *Babesia caballi* (**a**) and *Theileria equi* (**b**) at 72 h post-treatment

After demonstrating that TFQ has an inhibitory effect against both *T. equi* and *B. caballi*, we next evaluated the drug potency and drug response. The mean and range of IC_50_ values were calculated to compare the in vitro potency of TFQ against *B. caballi* and *T. equi* (Table 1; Fig. 5). TFQ exhibited approximately threefold greater potency against *T. equi* than against *B. caballi*, indicating species-specific differences in drug susceptibility. Notably, the IC_50_ values of TFQ against *T. equi* exhibited a narrower 95% confidence interval (CI), indicating a more consistent response across replicates. In contrast, the broader IC_50_ range observed for *B. caballi* may reflect greater biological variability or differences in assay sensitivity. The remarkable difference between IC_50_ and IC_99_ values against *T. equi* suggests a steep dose–response curve, whereas the relatively smaller difference against *B. caballi* indicates a more gradual inhibition profile.

**Table 1:** Concentrations of tafenoquine succinate that reduce cell viability of *Theileria equi* and *Babesia caballi* by 50% and 99%, respectively, after 72 h in in vitro cultures

Parasite	IC_50_ (μM)^a^	IC_99_ (μM)^a^
*Theileria equi*	5.904 μM (4.99–5.96)	60.74 μM (37.41–113.3)
*Babesia caballi*	14.5 μM (13.81–15.23)	20.44 μM (17.77–28.84)

*IC*_*50*_ Concentration that reduces cell viability by 50%,* IC*_*99*_ concentration that inhibits cell viability by 50%

^a^Values in parentheses are the 95% confidence interval

**Fig. 6:**
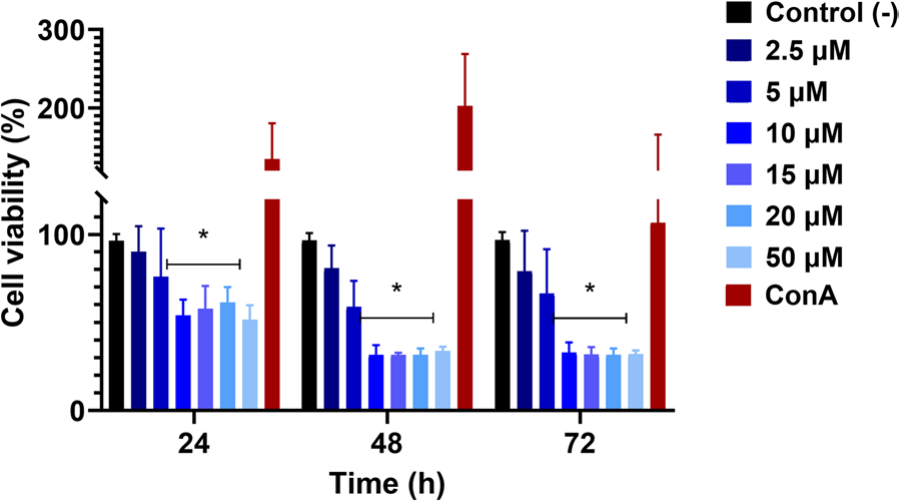
Peripheral blood mononuclear cell viability (%) at various TFQ concentrations measured in triplicate at 24-, 48- and 72-h post-treatment. Group columns marked with an asterisk indicate statistically significant differences (*P* < 0.05) compared to unmarked columns. ConA, Concanavalin A; TFQ, tafenoquine succinate

Finally, to assess compound selectivity and potential therapeutic windows, we measured cytotoxicity in mammalian cell lines and subsequently derived selectivity indices for each treatment. To evaluate the potential cytotoxic effects of TFQ on host cells, equine PBMCs were exposed to increasing concentrations of the drug, and PBMC viability was measured at 24, 48 and 72 h post-treatment. For the 24 h time point, the mean viability values were 100.0%, 100.0%, 76.8%, 54.0%, 57.9%, 61.4% and 51.6% for 0, 2.5, 5, 10, 15, 20 and 50 µM TFZ, respectively. A Friedman test indicated significant differences among concentrations (*χ*^2^ = 16.61, *P* = 0.015). Post hoc Dunn comparisons against the 0 µM control showed that viability at 2.5 µM (*P* > 0.05) and 5 µM (*P* > 0.05) were not significantly different from that of the control, whereas viability at 10 µM (*P* = 0.004), 15 µM (*P* = 0.006), 20 µM (*P* = 0.009) and 50 µM (*P* = 0.003) were significantly lower. These differences are also illustrated in Fig. 6, where group columns connected by an asterisk (horizontal line) are not significantly different from each other.

A similar pattern was observed at 48 and 72 h, with higher TFQ concentrations significantly reducing PBMC viability, while lower concentrations (≤ 5 µM) had no significant effect. These results indicate that TFQ at concentrations corresponding to the IC_50_ for *T. equi* do not compromise host cell viability. In contrast, cytotoxicity may be more relevant for *B. caballi*, whose IC_50_ is approximately threefold higher. Stimulation with ConA confirmed assay sensitivity, showing a significant increase in PBMC proliferation at 24 and 48 h (*P* < 0.05). Finally, TFQ exhibited a selectivity index of 1.69 for *T. equi* and 0.69 for *B. caballi*, indicating greater selectivity for *T. equi* over host PBMCs (Table 2).

**Table 2:** Specific selective index for tafenoquine succinate against *Theileria equi* and *Babesia caballi*

Parasite	IC_50_ (µM)	CC_50_ (µM, equine PBMCs)	Specific selective index (SSI)
*Theileria equi*	5.9	10	1.69
*Babesia caballi*	14.5	10	0.69

* CC*_*50*_ Concentration that reduces host cell viability by 50%,* IC*_*50*_ concentration that inhibits parasite growth by 50%,* PBMC* peripheral blood mononuclear cells

Cytotoxicity was observed to be dose- and time-dependent, with significant reductions in PBMC viability at TFQ concentrations of ≥ 10 µM, and the effect becoming more pronounced at 72 h post-treatment (Fig. 6). Overall, these findings demonstrate that TFQ exhibits limited cytotoxicity at concentrations relevant for *T. equi* inhibition, with effects becoming apparent only at higher concentrations and longer exposure times.

## Discussion

The present study assessed the in vitro efficacy of TFQ against *Theileria equi* and *Babesia caballi*. Our findings show that TFQ exerted a marked inhibitory effect on parasite growth, particularly on *T. equi*, with suppression persisting even after treatment withdrawal, suggesting a potential sustained parasiticidal activity. While *B. caballi* also responded to TFQ, its sensitivity was comparatively lower than that of *T. equi*. Nonetheless, TFQ at a concentration of 15 µM significantly reduced *B. caballi* parasitemia by day 3, with sustained suppression observed throughout the study period. Notably, *B. caballi* cultures treated with ID showed recrudescence after initial inhibition, whereas those treated with TFQ did not, suggesting that TFQ may be more effective in preventing parasite recovery.

Although the mechanism of action of TFQ remains unclear, a recent study using human primary hepatocytes demonstrated that primaquine, an 8-aminoquinoline closely related to TFQ, exerts its anti-plasmodial effect through a mechanism dependent on cytochrome P450 NADPH:oxidoreductase, accompanied by the production of hydrogen peroxide (H_2_O_2_) [33]. Given the structural relatedness between primaquine and TFQ, it is plausible to assume that the latter compound also acts through host-generated oxidative intermediates. While the in vitro system used in this study may not fully reflect the profile of reductases present in vivo, NADPH reductases are known to be present in erythrocytes, the cells used to support the growth of *T. equi* and *B. caballi* in culture [34]. Therefore, it is plausible to speculate that these enzymes could be responsible for generating host-derived oxidative intermediates in the presence of TFQ, which may explain the compound’s parasiticidal effect.

The IC_50_ for *T. equi* and *B. caballi* are 5.9 µM and 14.5 µM, respectively, which are values that technically fall within the broad *Plasmodium* range, but the implication of "comparable sensitivity" is misleading, especially as it is known that *P. falciparum* can be sensitive at submicromolar levels (e.g. 0.5 µM) [35, 37].

A critical aspect in comparing drug efficacy between species lies in analyzing the relationship between IC_50_ (concentration for 50% inhibition) and IC_99_ (near-complete inhibition). The gap between IC_50_ and IC_99_ offers insight into the compound’s dose–response dynamics. A wide gap suggests a steep dose–response curve, potentially indicating cooperative or heterogeneous mechanisms, whereas a narrow gap implies a more uniform and predictable response [36]. In this study, *T. equi* had the wider IC_50_–IC_99_ gap (IC_50_: 5.9 µM; IC_99_: 60.7 µM), indicating a steep dose–response curve. This pattern suggests that while low concentrations of TFQ partially suppress the parasite, much higher concentrations are required for near-complete clearance. Such steep curves may reflect heterogeneous susceptibility within the parasite population, cooperative drug interactions or differences in drug uptake or metabolism [15, 37]. A similar trend was observed in a previous study with novobiocin, where *T. equi* displayed a steeper curve than *B. caballi* [7]. The relatively narrower IC_50_–IC_99_ gap of *B. caballi* (IC_50_: 14.5 µM; IC_99_: 20.44 µM) indicates a more gradual and linear response to increasing TFQ concentrations, possibly indicating a more homogeneous parasite population or a more consistent interaction between the drug and its target [36, 38]. Similarly, narrow-gap profiles have been observed in other protozoan species with uniform drug sensitivity—such as clinical isolates of *Plasmodium falciparum* [39]- where limited subpopulation variability affected treatment outcomes. One may speculate that the wide confidence interval observed in the *T. equi* IC_99_ (Table 1) may be likely due to methodological variability rather than biological heterogeneity. Potential sources of variability may include the limited number of replicates, differences in parasite inoculum or measurement precision in parasitemia quantification. This variability could be minimized in future experiments by increasing the number of replicates and performing additional independent experiments, which would allow for more precise IC_99_ estimates. Despite the broad interval, the data clearly demonstrates dose-dependent inhibition by TFQ.

Interestingly, *B. caballi* has often exhibited greater sensitivity (lower IC_50_ values) to other drugs than *T. equi*, with varying data on the IC_50_–IC_99_ gap [40]. The findings in the current study suggest that although *B. caballi* exhibits a higher IC_50_ to TFQ, its narrower IC_50_–IC_99_ gap may allow more straightforward dosing strategies. In contrast, the broader range observed in *T. equ*i requires careful dose optimization to ensure complete parasite clearance.

TFQ demonstrated no significant cytotoxicity in equine PBMCs at concentrations up to 5 µM. Cell viability began to decline only at concentrations ≥ 10 µM (*P* < 0.05), which falls within or slightly above the effective antiparasitic range for *T. equi*, but remained below the concentration required for complete inhibition of *B. caballi*. The SSI of TFQ was nearly twice as high for *T. equi* as for host cells. In contrast, the SSI for *B. caballi* was only 0.69, indicating that the therapeutic concentrations required to inhibit this species either approach or exceed the cytotoxic threshold in PBMCs. These findings highlight TFQ’s more favorable safety margin and selectivity against *T. equi*, while also underscoring its narrower therapeutic window for *B. caballi*. It is important to note that although the SSI of 1.69 against *T. equi* falls below the threshold typically desired for lead optimization, it still provides a meaningful indication of differential activity and supports TFQ’s potential as a starting point for further structural refinement or combination strategies. Collectively, this underscores that the SSI should be interpreted in conjunction with other pharmacological parameters and the established mechanisms of action of TFQ. This profile is consistent with a previous study in human THP-1 cells, where TFQ demonstrated a relatively high median lethal dose (LD_50_) of 53.57 µM, indicating its preferential toxicity toward parasites over host cells [41]. Although TFQ has been associated with hemolytic toxicity in G6PD-deficient human erythrocytes [25], only a single case of G6PD deficiency has been reported in equines, unrelated to TFQ-induced hemolysis [40]. This suggests that although G6PD deficiency may have limited clinical relevance in horses, its significance remains uncertain and warrants further investigation.

Taken together, our results support the potential of TFQ as a promising alternative to ID for the treatment of EP, particularly for *T. equi*. Its sustained activity, low micromolar efficacy and low cytotoxicity make it a compelling candidate for further evaluation. Notably, TFQ’s broader antiparasitic spectrum and unique dose–response dynamics provide an opportunity for controlling mixed or resistant infections. While this study demonstrates the in vitro inhibitory activity of TFQ against *T. equi* and *B. caballi*, several limitations should be noted. The use of in vitro culture systems restricts direct extrapolation to in vivo conditions, where host immunity, drug metabolism and pharmacokinetics may affect treatment outcomes. Moreover, the mechanism of action of TFQ was not experimentally investigated, and cytotoxicity was assessed only in equine PBMCs. Therefore, further in vivo studies are required to evaluate the pharmacokinetics, safety and therapeutic efficacy of tafenoquine in horses.

## Conclusions

This study provides the first evidence of TFQ’s potent in vitro activity against *T. equi* and moderate activity against *B. caballi*, along with a mild cytotoxic profile in equine PBMCs. These findings lay the foundation for further preclinical and clinical research and support further validation as a novel chemotherapeutic option for equine piroplasmosis.

## Data Availability

All data generated or analyzed during this study are included in this article.

## Abbreviations

CC_50_: Concentration that reduces host cell viability by 50%
DMSO: Dimethyl sulfoxide
EP: Equine piroplasmosis
FDA: US Food and Drug Administration
G6PD: Glucose-6-phosphate dehydrogenase
IC_50_: Half-maximal (50%) inhibitory concentration
IC_99_: 99% Inhibitory concentration
ID: Imidocarb dipropionate
PBMCs: Peripheral blood mononuclear cells
TFQ: Tafenoquine succinate
